# Functional frontal lobectomy in the surgical treatment of pharmacoresistant frontal lobe epilepsy: how I do it

**DOI:** 10.1007/s00701-024-06176-x

**Published:** 2024-07-18

**Authors:** Francesca Battista, Alice Esposito, Giovanni Muscas, Alessandro Della Puppa

**Affiliations:** 1 Department of Neurosurgery, Department of Neuroscience, Psychology, Drug Area and Child Health (NEUROFARBA), University of Florence, Careggi University Hospital, Florence, Italy; 2 University of Florence, School of Human Health Sciences, Firenze, Italia

**Keywords:** Frontal lobectomy, Pharmacoresistant, Epilepsy

## Abstract

**Background:**

Frontal lobe epilepsy is pharmacoresistant in 30% of cases, constituting 10–20% of epilepsy surgeries. For cases of no lesional epilepsy (negative MRI), frontal lobectomy is a crucial treatment, historically involving Frontal Anatomical Lobectomy (AFL) with a 33.3% complication risk and 55.7% seizure control.

**Methods:**

We describe Frontal Functional Lobectomy (FFL), in which the boundaries are defined on the patient's functional cortico-subcortical areas, recognized with advanced intraoperative technologies such as tractography and navigated transcranial magnetic stimulation (nTMS).

**Conclusions:**

The FFL allows for a broader resection with a lower rate of postoperative complications than the AFL.

**Supplementary Information:**

The online version contains supplementary material available at 10.1007/s00701-024-06176-x.

## Manuscript

### Relevant surgical anatomy

The frontal lobe is the largest of the cerebral lobes and houses fundamental functions such as language and movement. It comprises a lateral, a mesial, and a basal surface, separated posteriorly from the parietal lobe by the central sulcus and defined anteriorly by the frontal pole.

**Fig. 1:**
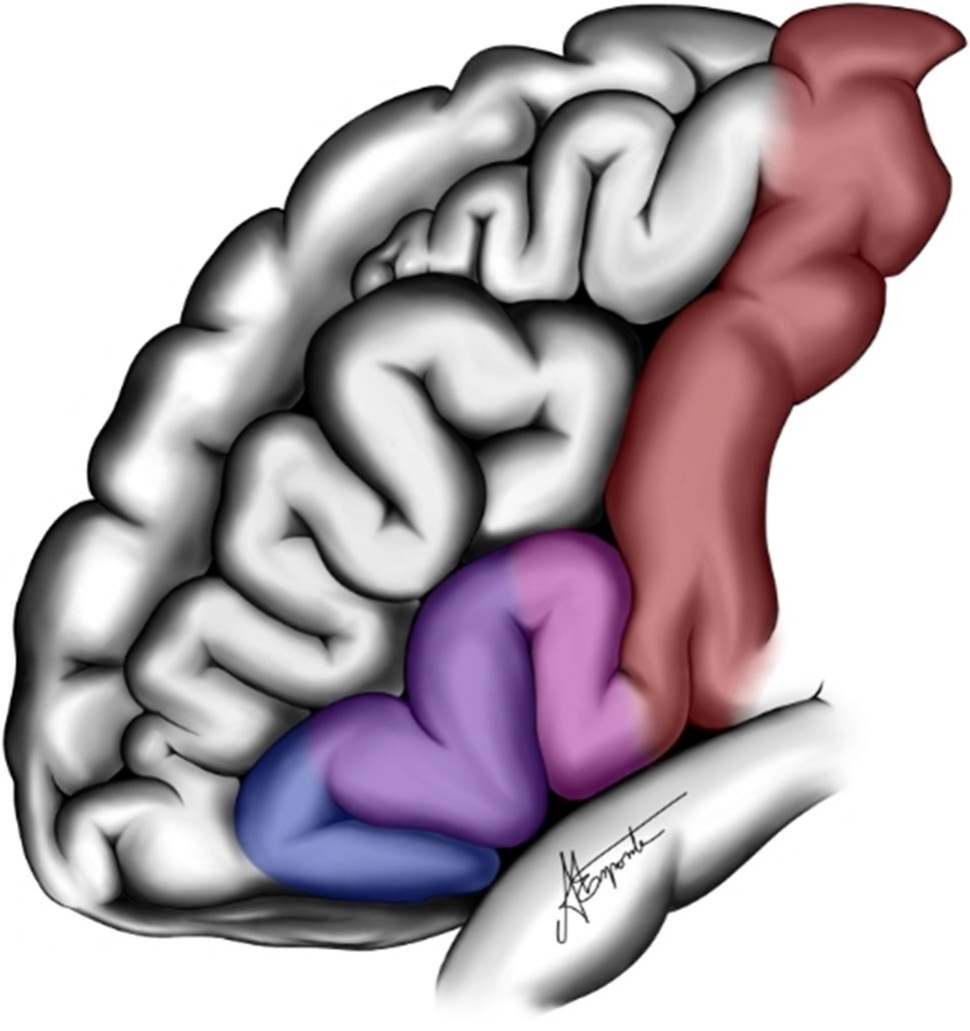
Lateral surface of frontal lobe: In red, the primary motor area; in pink, the pars opercularis; in purple, the pars triangularis; and in blue, the pars orbitalis of the inferior frontal gyrus (F3)

Its lateral surface features the precentral gyrus, represented by the primary motor area, located just anterior to the central sulcus (Fig. 1). The remaining portion of the lateral surface is divided by two longitudinally oriented sulci: the superior frontal sulcus and the inferior frontal sulcus, which divide the superior, middle, and inferior frontal gyri. The inferior frontal gyrus is subdivided into three parts known as pars orbitalis, pars triangularis, and pars opercularis, described in an anteroposterior direction.

**Fig. 2:**
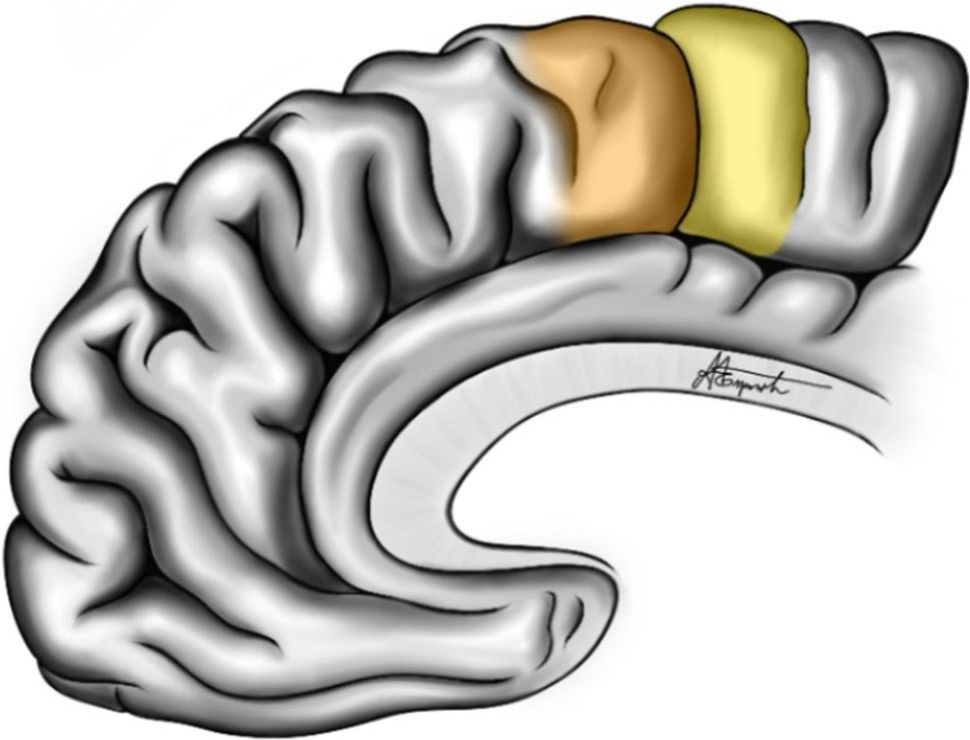
Medial surface of frontal lobe: In yellow, the supplementary motor area (SMA), and in orange, the pre-SMA

The medial face of the frontal lobe presents the anterior portion of the paracentral lobule, the medial continuation of the primary motor cortex (Fig. 2). Anterior to this lies the supplementary motor area (SMA), and anterior to that is the pre-SMA. The lower boundary of these three areas is the cingulate sulcus, which separates them from the cingulate gyrus below (anterior and middle portions), following the course of the corpus callosum.

**Fig. 3:**
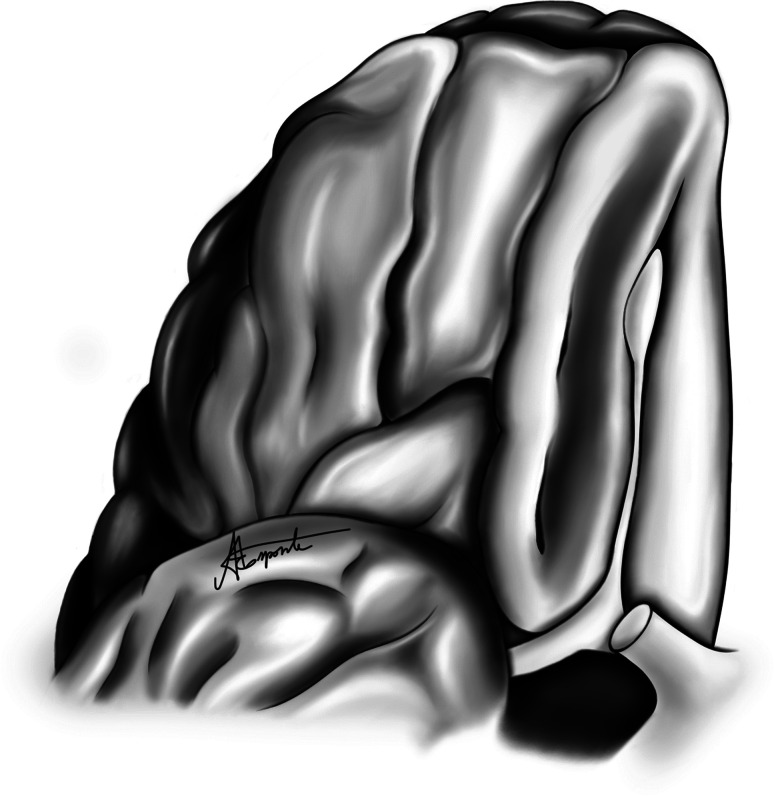
Inferior surface of frontal lobe

**Fig. 4:**
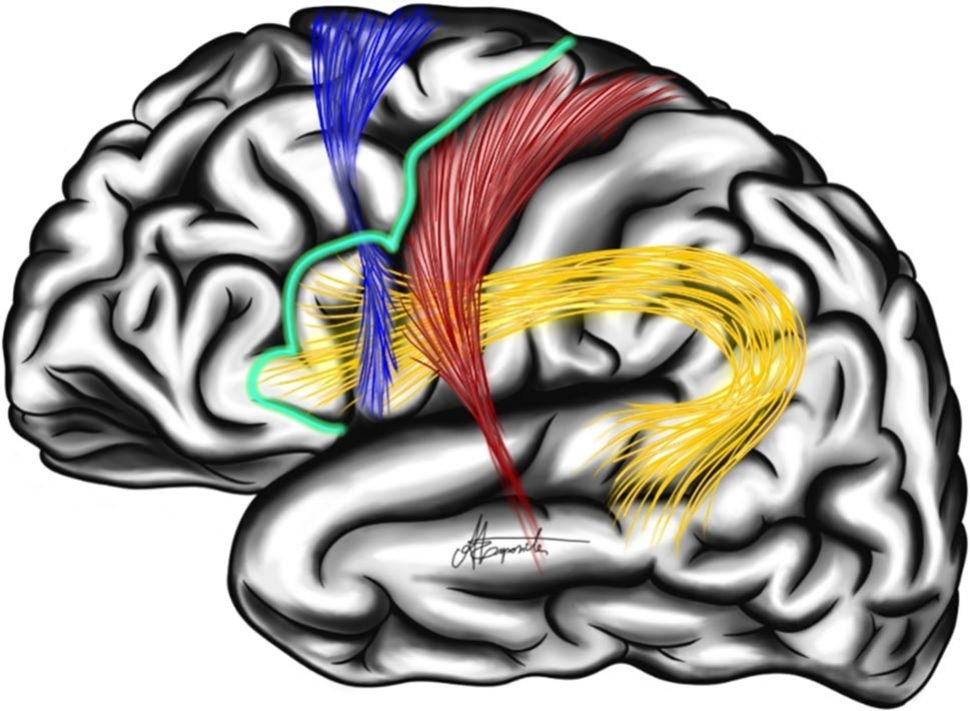
Representation of the lateral surface of the brain, highlighting the arcuate fasciculus (yellow), the ASLANT (blue), and the pyramidal tract (red). The posterior boundary of the functional frontal lobectomy (FFL) resection is marked by the blue edge, which is located near the ASLANT and the pyramidal tract, identified through intraoperative subcortical stimulation

The basal surface features the rectus gyrus medially, separated by the olfactory sulcus from the lateral portion housing the olfactory bulb. The lateral part is divided by the orbital sulcus into an H shape. The medial orbital gyrus lies closest to the olfactory sulcus. The middle part forms H center split into anterior and posterior orbital gyri. The lateral orbital gyrus is outermost (Fig. 3). Regarding the frontal lobe's white matter, the pyramidal tract is formed from the primary motor cortex following a somatotopic organization of body parts. In the posterior portion of the inferior frontal gyrus, the anterior part of the arcuate fasciculus is found, extending to the pars triangularis and posteriorly to the supramarginal gyrus of the parietal lobe and then to the superior, middle, and inferior temporal gyri. The ASLANT tract develops from the SMA, extending to the inferior frontal gyrus (Fig. 4).

### Description of the technique

Functional frontal lobectomy (FFL) is a lengthy and demanding procedure for the patient; therefore, we do not perform it in awake surgery. The limits of the resection are defined mainly by the functional anatomy of the specific patient. The posterior margin of lobectomy is immediately anterior to the primary motor area. To identify the cortical motor area, perform the preoperative transcranial magnetic stimulation (TMS) and then transfer data onto the Neuronavigator (StealthStation 8, Medtronic) to identify them on the cortex. We then confirm these points with direct cortical stimulation (DCS) in cortical mapping, representing the first surgical phase performed using the train-of-fivetechnique [5]. The train-of-five technique, described by Taniguchi in 1993, consists of a train of 5 pulses, 0.5-ms pulse width each, at a frequency of 250 Hz, equivalent to an interstimulus interval of 4 ms [5]. Once the primary motor area is identified, we proceed with resection along the anterior sulcus to this area, with constant monitoring of motor function conducted using motor-evoked potentials (MEPs) performed transcortically and cortically by placing a strip along the motor area.

**Fig. 5:**
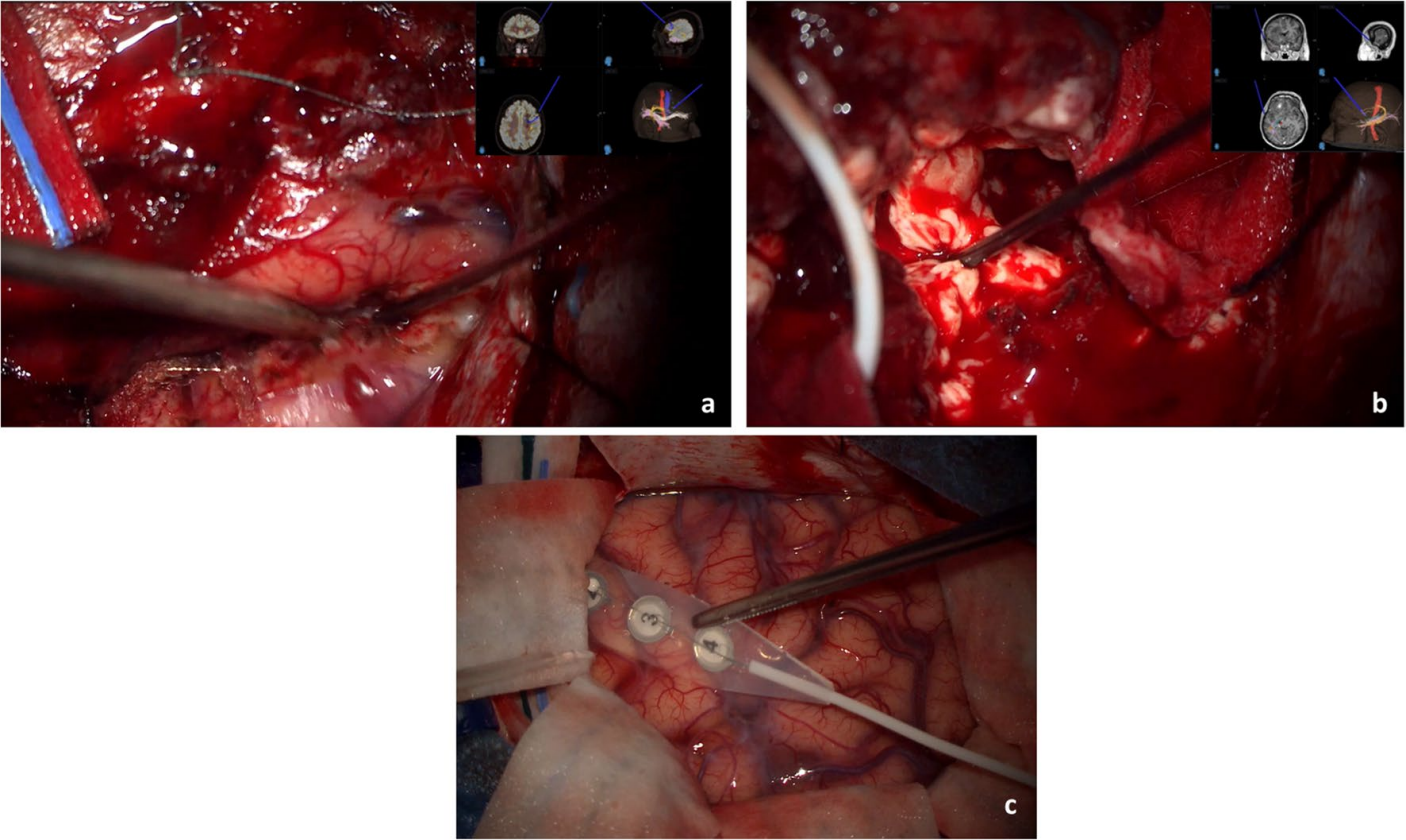
**a**. Identification of corticospinal tract with tractography to define the posterior margin of FFL; **b**. Identification of arcuate fasciculus with tractography to define the posterior margin of FFL. FFL: functional frontal lobectomy. 5c. Positioning of the strip in the posterior temporal lobe to register CCEP to monitor the integrity of arcuate fasciculus during resection. CCEP: cortico-cortical evoke potentials

The depth limit of the lateral margin of the resection is behind the corticospinal tract, which is identified in the tractography on Neuronavigation (Fig. 5a, b). We also perform subcortical direct stimulation during the resection to define the distance to the corticospinal tract. We adopt 5 mm as the minimum distance to maintain.

In the initial lateral frontal lobe resection we preserve the inferior frontal and orbital gyrus, that is crucial in surgeries on the dominant side. The posterior limit of resection on the inferior frontal gyrus is defined with the help of cortico-cortical evoked potentials (CCEP), which are used to monitor the integrity of the arcuate fasciculus [4] (Fig. 5c). A stimulation strip is placed in the inferior frontal gyrus, and the recording strip in gyri T1-T3 (Fig. 5c). A cortical evoked potential is recorded in the recording strip following low-frequency stimulation on the stimulating strip. The stimulation is repeated during surgical excision to monitor the functionality of the arcuate fasciculus [4].

Resection of the rectal and orbital gyrus follows, with the lateral limit established just in front of the lesser wing of the sphenoid. The olfactory trigone is a landmark for the posterior limit of frontobasal removal, necessitating complete removal of the basal surface until just anterior to the olfactory trigone through subpial aspiration [2]. The depth limit of resection is the corpus callosum.

We assessed the quality of the FFL by an early postoperative MRI and EEG. We followed up on the patients one year with serial EEGs and clinical follow-ups to assess the Engel class.

### Indications

Frontal lobectomy is the predominant surgical intervention for non-lesional pharmacoresistant frontal lobe epilepsies (FLE) [2]. It was reported that the seizure outcome for FLE (45–60%) is generally unsatisfactory in comparison with those of temporal lobe epilepsy [3], and this is probably because of the absence of an anatomical target. In a recent meta-analysis, it was shown that FLE patients with focal and identifiable lesions are more likely to achieve seizure freedom than those with a more poorly defined epileptic focus after frontal corticectomy [1]. For this reason, FFL is the primary technique in the case of FLE without evident lesions.

### Limitations

The main limitation of this technique is represented by ventricular opening, an event also observed in anatomical frontal lobectomy (AFL). Ventricular opening is associated with the risk of postoperative hydrocephalus, which often necessitates a second surgical intervention, frequently involving the implantation of permanent external devices (e.g., ventriculoperitoneal shunt). However, ventricular opening confers the advantage of achieving the broadest possible frontal lobectomy within functional limits, increasing the likelihood of success regarding epileptological outcomes. A technique of frontal decortication with preservation of the frontal horn has been described [6]. However, this technique is associated with less epilepsy control and reduced applicability in cases where the ventricular horn is poorly surrounded by white matter, as in some epileptic pathologies [6].

Another limitation of FFL is the low risk of postoperative transitory motor and language deficits.

### How to avoid complication

**Fig. 6:**
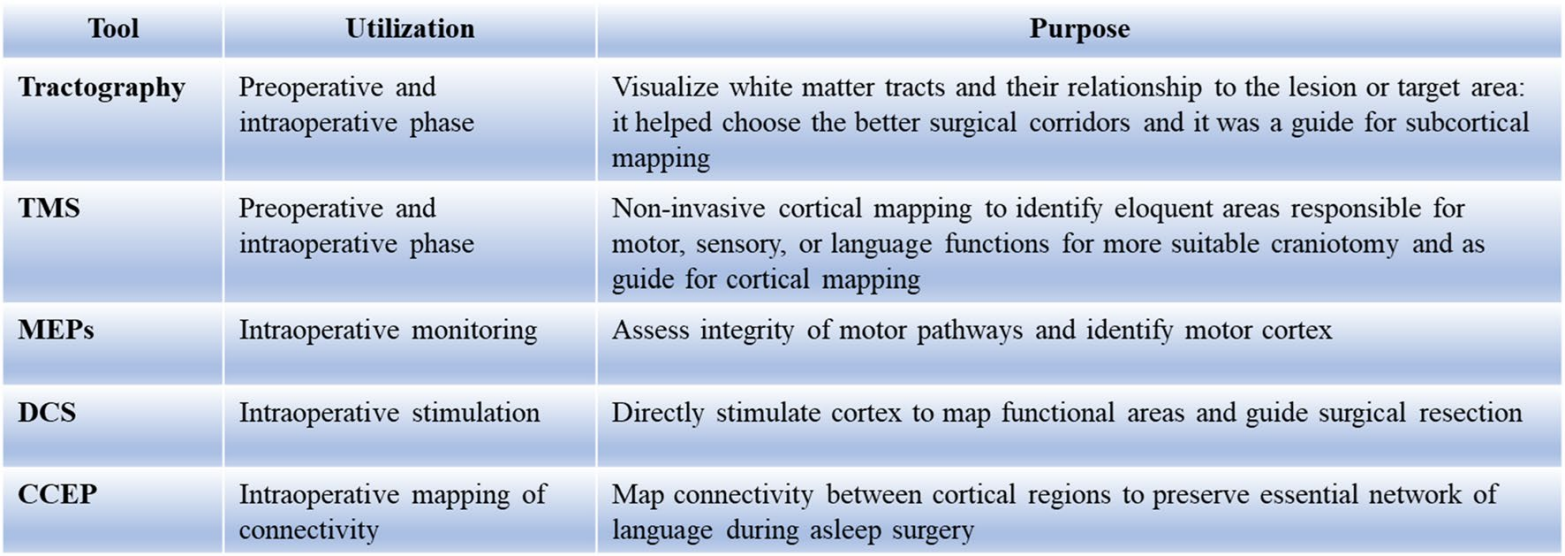
Utilization of different techniques before and during surgery for predicting the localization and mapping of eloquent areas and cortical tracts. Techniques include tractography, transcranial magnetic stimulation (TMS), motor evoked potentials (MEPs), direct cortical stimulation (DCS), and cortico-cortical evoked potentials (CCEP)

The most common technique adopted for FLE is AFL, but this is burdened by a 33.3% complication rate and epilepsy control in 55.7% of cases [2]. FFL is indicated in all cases of frontal lobectomy, with the advantage of having fewer complications than AFL and equal epileptological outcomes. The lowest rate of postoperative neurological deficits is attributed to the use of advanced pre- and intraoperative technologies such as TMS, tractography, DCS, and CCEP, which allow the identification of the location of specific functional structures in the patient and also enable monitoring of their integrity throughout the duration of the lobectomy, thereby minimizing the risk of permanent deficits. All tools used are listed in Fig. 6. In our experience with FFL, no patient has experienced permanent deficits postoperatively, unlike AFL.

### Specific information for the patient

The patient must be informed of the possibility of postoperative neurological deficits, with transient deficits being more likely than permanent ones (e.g., hemiparesis or aphasia). They should be informed of the risk of hydrocephalus and the potential need for a second surgery. Additionally, they should be informed of the possibility of incomplete resolution of epileptic seizures.

## Supplementary Information

Below is the link to the electronic supplementary material.

Supplementary file1 (MP4 188550 KB)

## Data Availability

Not applicable.
